# Biosignal Analysis to Assess Mental Stress in Automatic Driving of Trucks: Palmar Perspiration and Masseter Electromyography

**DOI:** 10.3390/s150305136

**Published:** 2015-03-02

**Authors:** Rencheng Zheng, Shigeyuki Yamabe, Kimihiko Nakano, Yoshihiro Suda

**Affiliations:** 1 Institute of Industrial Science, The University of Tokyo, Tokyo 153-8505, Japan; E-Mail: suda@iis.u-tokyo.ac.jp; 2 New Industry Creation Hatchery Center, Tohoku University, Sendai 980-8579, Japan; E-Mail: yamabe@niche.tohoku.ac.jp; 3 Interfaculty Initiative in Information Studies, The University of Tokyo, Tokyo 153-8505, Japan; E-Mail: knakano@iis.u-tokyo.ac.jp

**Keywords:** biosignal, electromyography, masseter, mental stress, palmar perspiration

## Abstract

Nowadays insight into human-machine interaction is a critical topic with the large-scale development of intelligent vehicles. Biosignal analysis can provide a deeper understanding of driver behaviors that may indicate rationally practical use of the automatic technology. Therefore, this study concentrates on biosignal analysis to quantitatively evaluate mental stress of drivers during automatic driving of trucks, with vehicles set at a closed gap distance apart to reduce air resistance to save energy consumption. By application of two wearable sensor systems, a continuous measurement was realized for palmar perspiration and masseter electromyography, and a biosignal processing method was proposed to assess mental stress levels. In a driving simulator experiment, ten participants completed automatic driving with 4, 8, and 12 m gap distances from the preceding vehicle, and manual driving with about 25 m gap distance as a reference. It was found that mental stress significantly increased when the gap distances decreased, and an abrupt increase in mental stress of drivers was also observed accompanying a sudden change of the gap distance during automatic driving, which corresponded to significantly higher ride discomfort according to subjective reports.

## 1. Introduction

The development of intelligent vehicles that can drive automatically is an exciting advance that might change people’s daily life [1,2]. Automatic driving technology can be also applied to maintain close gap distances between multiple trucks, to reduce air resistance for low fuel consumption and high traffic density [3]. Related studies have indicated that the air resistance can be reduced by about 8%, 12%, and 17%, for gap distances of 12, 8, and 4 m, respectively [4,5]; however, the drivers may experience high levels of mental stress. In fact, an objectively ergonomic assessment is critically important for this case but it was neglected by the past research [6,7].

An objective assessment of mental stress is based on inferring psychological significance from physiological signals, namely biosignals [8]. For instance, as a wearable measurement, electroencephalography (EEG) and electrocardiography (ECG) can provide real-time stress detection, although both techniques always require complicated signal processing [9,10,11,12]. An electromyography (EMG) signal can indicate a continuous mental stress estimation [13]. The EMG signals of the trapezius and sternocleidomastoid muscles had been applied as predictors of psychological and physiological stresses; however, the two muscles are more or less involved in driving operations and motions [14,15]. To resolve this problem, masseter EMG signals can be adopted to observe mental stress-induced changes [16,17]. In addition, there are thousands of published contributions on the study of mental stress indices, which were mainly concentrated on the application of the physiological signals like skin temperature, respiratory measures, blood pressure, EEG, and ECG [18,19,20,21].

As an important physiological signal, perspiration has medical, pharmaceutical, biochemical, and psychological implications. Active perspiration induced by mental stress can occur over the whole surface of the skin but is usually confined to the palms, soles of the feet, axillae, and forehead. Despite the differences in its response to stimuli, there is little evidence that palmar perspiration functions differently from those produced by other positions [22,23]. Importantly, by application of ventilated capsule method with a capacitive thin-film humidity sensor, a wearable perspiration rate meter had been developed for continuous and accurate measures of palmar perspiration [24,25]. However, further innovative study was still few for deeper understanding of emotional or mental reactions by application of this wearable sensor system.

On the other hand, owing to safety and ethical issues, it has not been possible to investigate the mental stress of drivers in an actual-vehicle driving condition. In this case, driving simulators are efficient and reliable tools for studying human factors in a repeatable driving condition [26]. To provide a high-realistic sensation closed to real world scenarios, an advanced driving simulator was significantly improved by modifying and adjusting the visual system, rotation center, and sound generation system [27]. By application of the driving simulator, emergent avoidance behaviors were investigated in automatic driving of trucks [28]. The current study has extended the former research to quantitatively assess mental stress of drivers engaged in automatic driving of trucks using two distinct biosignals: palmar perspiration and masseter EMG signals.

This paper starts with a description of automatic driving of trucks in the driving simulator; then, biosignal measurement and processing are presented related to palm perspiration and masseter EMG signals. In the following section, the experimental contents are demonstrated including participants, protocol, and data collection. After the interpretation of results, conclusions are provided in the last section.

## 2. Materials and Methods

### 2.1. Automatic Driving of Trucks

**Figure 1:**
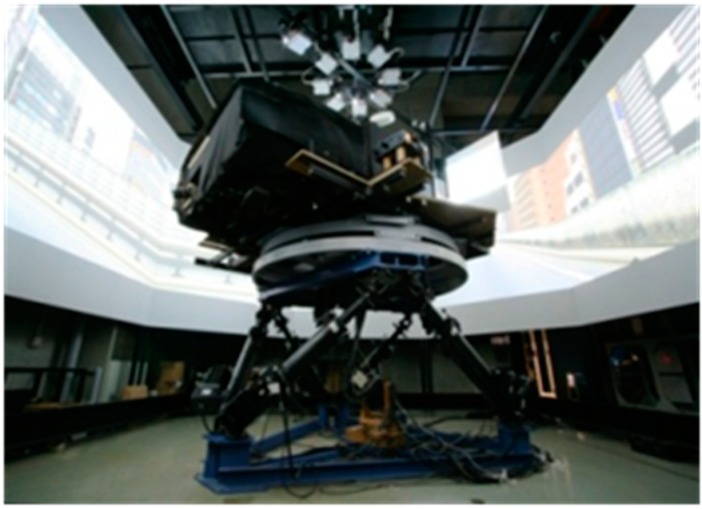
Photograph of the driving simulator.

A highly realistic driving simulator was used to generate an experimental scenario of automatic driving of trucks. As shown in Figure 1, the driving simulator included a 360-degree screen showing the view from inside a vehicle with two side mirrors, and a 6-degrees-of-freedom moving platform with maximum acceleration of 4.9 m/s^2^. The truck dynamics were realized by integrating TruckSim (Mechanical Simulation, Ann Arbor, MI, USA) and the real-time dSPACE system (dSPACE, Paderborn, Germany). The libraries of dSPACE and TruckSim were overlapped using the Simulink library (MathWorks, Natick, MA, USA). Furthermore, a control algorithm by Simulink for gap distance control was designed to create automatic driving, and the controlling signals for braking, accelerating, and steering were taken from the TruckSim connecting with the driving simulator system.

In this control algorithm, *x_i_*, *V_i_*, and *u_i_* represent the position, velocity, and acceleration of the *i*-th truck. When a value for acceleration inputs, the motion for the vehicle can be expressed by:

(1) $$\left[\begin{array}{c} {\dot{x}}_{i}\\ {\dot{V}}_{i} \end{array}\right]=\left[\begin{array}{cc} 0 & 1\\ 0 & 0 \end{array}\right]\left[\begin{array}{c} {\dot{x}}_{i}\\ {\dot{V}}_{i} \end{array}\right]+\left[\begin{array}{c} 0\\ 1 \end{array}\right]u_{i}$$

The gap distance error *e_i_* and velocity error *ω_i_* can be defined as:

(2) $$e_{i}=x_{i}-x_{i+1}-L-d$$

(3) $$\omega_{i}=V_{i}-V_{ir}$$

where *L* is the length of the truck, *d* is the target value of the gap distance, and *V_ir_* is the target velocity of the *i*-th truck. In this study, the target velocity of the trucks was 80 km/h, but the target gap distances were 4, 8, and 12 m for the experimental setup. The preceding truck was projected on the screen, and the following truck was realized by the moving platform.

### 2.2. Biosignal Measurement

#### 2.2.1. Palmar Perspiration

**Figure 2:**
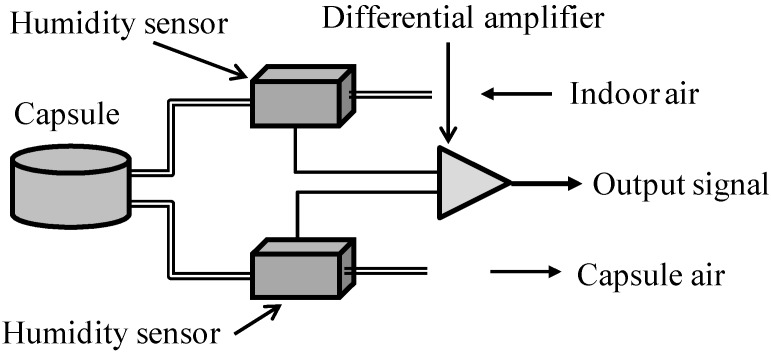
Schematic of the digital perspiration meter.

A digital perspiration meter was used to measure palmar perspiration, which includes a main body, two plastic probes, two resin capsules, and two capsule holders (SKN-2000, Nishizawa Electric Meter, Nakano, Japan). As shown in Figure 2, the portable meter uses a differential amplifier built into a micro-computer system to calculate the relative humidity of human skin compared with that of the atmosphere, and can records the perspiration rate continuously.

**Figure 3:**
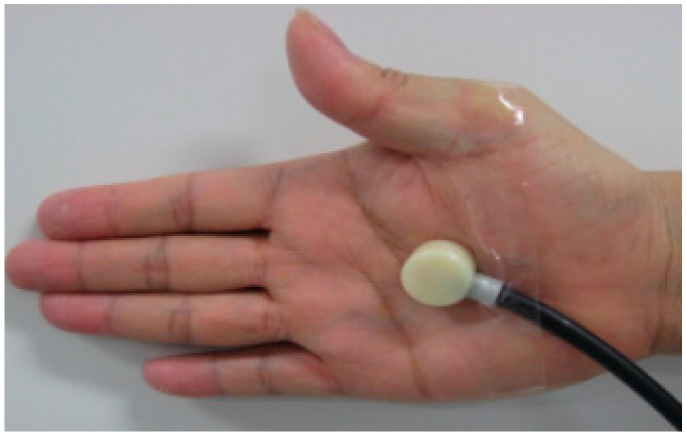
Palm with attached capsule.

During the experiment, the palmar perspiration sensor was positioned near the center of the palm in Figure 3. A double-sided sticky tape around the open-end of the capsule was used to attach the capsule to the central part of the palm. Then, a plaster cloth, 5 cm in width, was used to cover and tightly bind the capsule to the palm. Only one capsule was attached on right palm for each participant, thus it is presumed that the steering operations would not be affected. In the preliminary calibration, the step response for measuring the perspiration samples had a rise time of less than 1 s and a baseline fluctuation for 6 h of continuous measurements (without water intake) within 0.05 mg/cm^2^·min. The range of the measured perspiration rate was 0–4 mg/min with a flow control system built into the digital perspiration meter. The room temperature set at 23 °C with relative humidity at 60% HR.

#### 2.2.2. Masseter Electromyography

**Figure 4:**
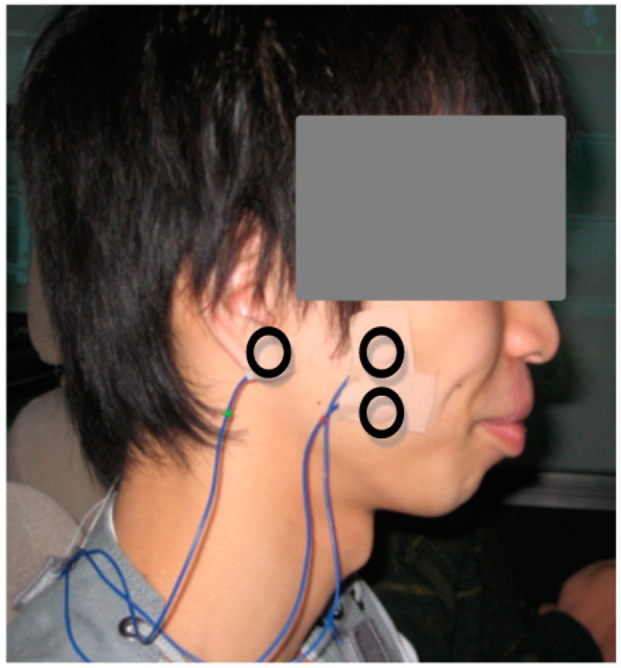
Right side of a participant’s head, showing the reference electrode attached to the ear lobe, and paired electrodes pasted on the right masseter muscle.

An active electrode (Teac, Tama, Japan), a conversion box (AP-U040, Teac), and amplifier-embedded collection equipment (Polymate AP1132, Teac) were used to record masseter EMG signals. Prior to applying the electrodes, the skin surface of the masseter muscle was cleaned using medical cotton gauze dipped in medical alcohol. Then, the electrodes were pasted to the skin surface of the masseter muscle. Earth and reference electrodes were pasted on the ear lobes. The positions of the masseter muscle electrodes are shown in Figure 4.

Before recording EMG signals, it was confirmed that the skin resistance at the masseter muscle was less than 100 Ω and the skin resistance at the reference and ground electrodes on the ear lobes was less than 50 Ω. In addition, it was ensured that the range of fluctuation of the skin resistance was less than ±10 Ω. The sampling frequency was 1000 Hz. In the pretreatment stage of data analysis, a high-pass filter at 10 Hz and a low-pass filter at 450 Hz were adopted to avoid motion artifacts and power-line interference.

### 2.3. Biosignal Processing

Through a biosignal processing design for the palmar perspiration rate and EMG activity of the masseter muscle, it is desired to compare psychophysiological responses to different conditions and events during the automatic driving, Thereby, a stress intensity index was proposed to evaluate the mental stress of each participant by the processes of rectification, smoothness, normalization, and evaluation of the collected biosignals.

Firstly, the collected biosignal was rectified and average rectified value (ARV) can be defined by:

(4) $$\mathrm{ARV}(t)=\frac{1}{2T}\int_{-T}^{T}\left|e(t+\tau )\right|\begin{array}{c} \text{d} \end{array}\tau$$

where e(t) is the biosignal, (−T,T) is the sampling range, and τ is the step length of sampling.

Then, a five-point moving average was applied to smoothen biosignals by:

(5) $$y_{i}=\frac{1}{2N+1}\sum_{n=-N}^{N}x_{i-n}\;\;\;\;\;\;\;\;\;\;\;\;\;\;\;(i=1,\;2,\;\cdots ,\;m)$$

where *y_i_*, *x*, *m*, and 2*N* + 1 are smoothened result, sampling data, data number, and average number. In here, *N* = 2 is for five-point moving average.

Normally, a maximum value of biosginal was adopted to deal with individual difference. However, it is difficult to measure EMG signal for maximum voluntary contractions or maximum value of the perspiration rate in this study. Thereby, gross average of the biosignal was applied to figure out individual characters and normalize the measured biosignals for all subjects [29]. The normalization of the bio-signals can be expressed by:

(6) $$y_{i}=\frac{nx_{i}}{\sum_{i=1}^{m}x_{i}},\begin{array}{ccc} & & \left(i=1,\;2,\;\cdots ,\;m\right) \end{array}$$

Root-mean-square value is normally used to evaluate variation of the measured signal; however, the root-mean-square method is conflicting with the above average normalization method. Referring fourth power method [30], root-mean-quad (RMQ) value is proposed for assessing mental effects of the automatic driving and provides an evaluation index which presents the cumulative stress intensity over time. In contrast to the root-mean-square value, the fourth power parameters have the advantage of not being limited to low crest factors of the biosignals. By their fourth power dependence on the biosginal level, they tend to emphasize more the effects of short duration events or variations, considered to have crucial stimulus on mental stress of participants. Mathematically, it is defined as:

(7) $$RMQ{\left(t\right)}_{SM}={\left(\frac{1}{T}\int_{0}^{T}e^{4}(t+\tau )\begin{array}{c} d \end{array}\tau \right)}^{\frac{1}{4}}$$

### 2.4. Experiment

#### 2.4.1. Participants

Ten healthy males were cooperated with driving experiments. The participants were 32.9 ± 12.9 years of age (Mean ± S.D.), 172.5 ± 6 cm in height, and weighed 63 ± 9 kg. The participants drove an average of 6.7 ± 5.4 times per month, and had 11.2 ± 10.9 years of driving experience. Prior to this driving experiment, the participants had driven the driving simulator an average of 8 ± 6.1 times; however, no participants had experience with automatic driving of trucks.

#### 2.4.2. Protocol

All participants received a detailed explanation about the purpose of the study before engaging in the experiment, and a legal agreement to cooperate in the driving experiments was approved by all the participants. For the preparation of the experiment, each participant was asked to operate the driving simulator and experience the automatic driving for about 20 min until they familiarized themselves with the driving environment. The driving experiment was then carried out for the four experimental conditions in the car-following modes: the manual driving condition in which participants were asked to keep a gap distance of 20–30 m from the preceding vehicle, and three automatic driving conditions with automatically set gap distances of 4, 8, and 12 m from the preceding vehicle.

The participants completed two experimental sessions. Both sessions included the manual driving trials first, followed by the automatic driving trials in randomized sequences with gap distances of 4, 8, and 12 m. Each experimental condition lasted about 5–8 min, with a 5 min rest after each condition was presented. The experimental condition, repetition, order, and duration are presented in Table 1.

**Table 1:** Experimental condition, repetition, order, and duration.

Experimental Condition	Repetition	Order	Duration
Manual driving with 20–30 m gap distance	twice	fixed	5–8 min
Automatic driving with 12 m gap distance	twice	randomized	5–8 min
Automatic driving with 8 m gap distance	twice	randomized	5–8 min
Automatic driving with 4 m gap distance	twice	randomized	5–8 min

In both manual and automatic driving, at the beginning of the trial, the preceding vehicle gradually accelerated to 80 km/h and then maintained this travel speed. However, after about 5 min driving, the preceding vehicle was programmed in a randomized place and time point to decelerate without notice from 80 to 30 km/h during a 2.5 min period. In the manual driving, it was necessary for the participants to brake to avoid a rear-end collision. In contrast to the manual driving, the driving simulator was also programmed to decelerate as a following vehicle while deceleration of the preceding vehicle. For whole automatic driving, the participants were not required to make any actions to operate the driving simulator, just to sit in the seat and look straight ahead.

#### 2.4.3. Data Collection

Driving parameters, palmar perspiration rate, and masseter EMG signals were measured during the whole driving experiment. The driving parameters were collected with 60 Hz sampling frequency and the palmar perspiration and EMG signal with 1000 Hz sampling frequency while the driver operated the driving simulator system. Meanwhile, a trigger signal was also input to ensure data synchronization. The stress intensity was calculated by application of the signal processing, for the palmar perspiration and EMG signals measured in for the four experimental conditions. Then, a factorial ANOVA was analyzed to know statistically significant differences of driver responses in the different experimental conditions.

Subjective evaluation was executed after each driving condition of the experiment. The participants were asked to complete a questionnaire about their ride comfort for the different driving conditions. One question was prepared for the drivers: “How do you rate your ride comfort for this time driving on a five-level scale?” There were five levels of ride comfort from which to select, and the five levels of evaluation were 1 = discomfort, 2 = a little discomfort, 3 = normal, 4 = a little comfort, and 5 = comfort.

## 3. Results and Discussions

### 3.1. Driving Parameters

**Figure 5:**
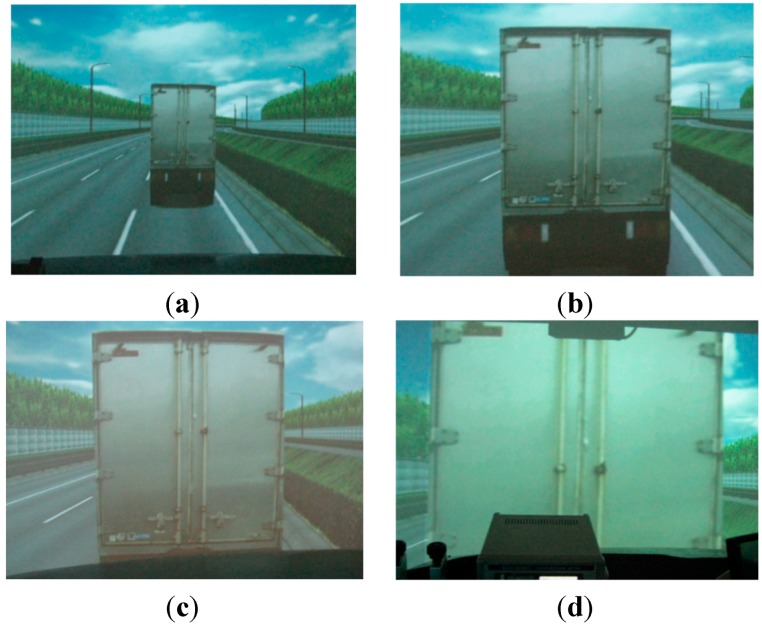
Preceding vehicles with the different gap distances. (**a**) 25 m gap distance; (**b**) 12 m gap distance; (**c**) 8 m gap distance; (**d**) 4 m gap distance.

On the hypothesis that the closer gap distance may result in higher mental stress of drivers in the automatic driving, four gap distances were prepared for this experimental study: 20–30 m gap distance as a reference for the manual driving, and 12, 8, and 4 m gap distances for automatic driving of trucks. In Figure 5, the preceding vehicles are presented with the visual angle of the drivers for the different gap distances.

**Figure 6:**
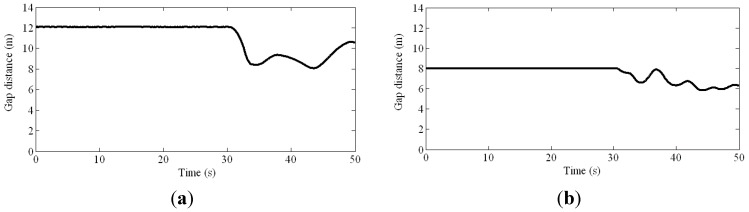

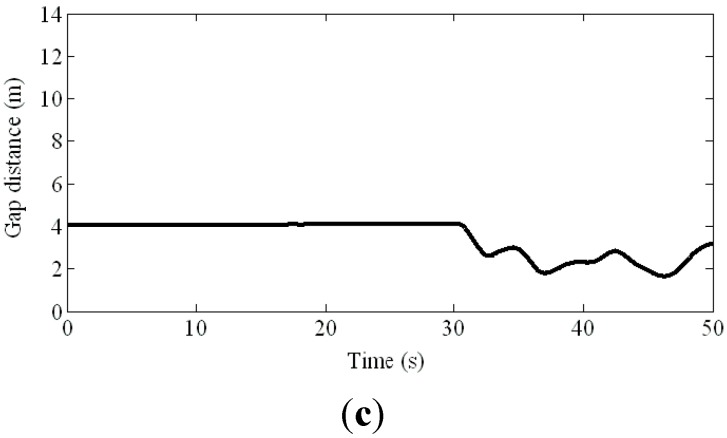
Variable gap distances in the automatic driving. (**a**) 12 m gap distance; (**b**) 8 m gap distance; (**c**) 4 m gap distance.

Furthermore, on the hypothesis that the variable gap distance may also result in higher mental stress of drivers than that of constant gap distance, same randomized deceleration of the preceding vehicle without notice was set for each manual and automatic driving. Correspondingly, the driving simulator made an automatically sudden braking in automatic driving to avoid rear-end collision. But the participants had to brake by themselves in the manual driving. In Figure 6, the example of the variable gap distances are presented in the automatic driving conditions. In this figure, 50 s time periods are picked out from the total 5–8 min driving, and decelerations began at the 30 s time point.

### 3.2. Biosignal Analysis

**Figure 7:**
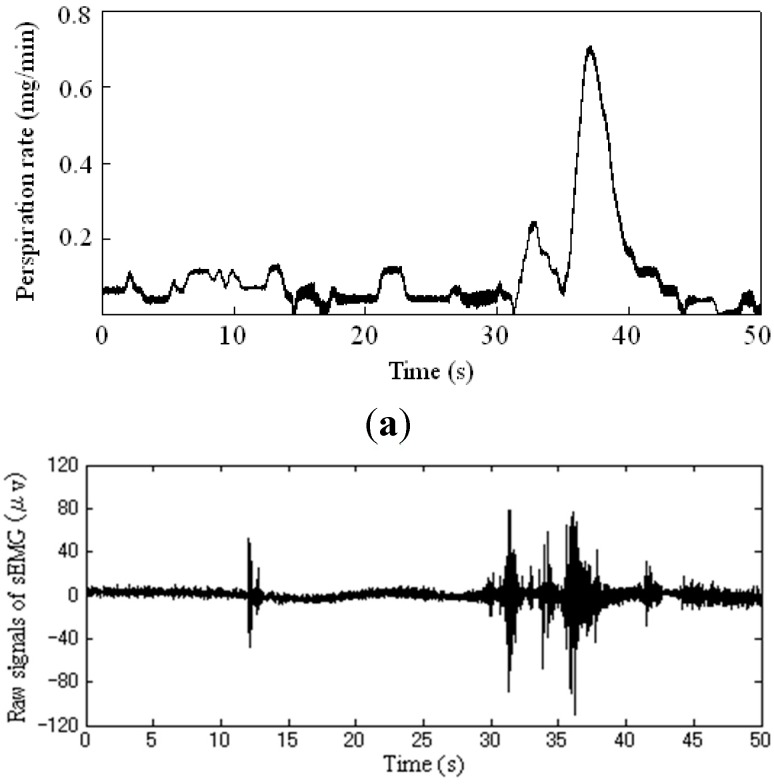
Examples of the measured biosignals in the automatic driving with a 4 m gap distance. (**a**) Raw signal of palmar perspiration rate; (**b**) Raw EMG signal of masseter.

The examples of the measured biosignals are presented in Figure 7, for the raw palmar perspiration and EMG signal of masseter in the automatic driving condition with a 4 m gap distance.

**Figure 8:**
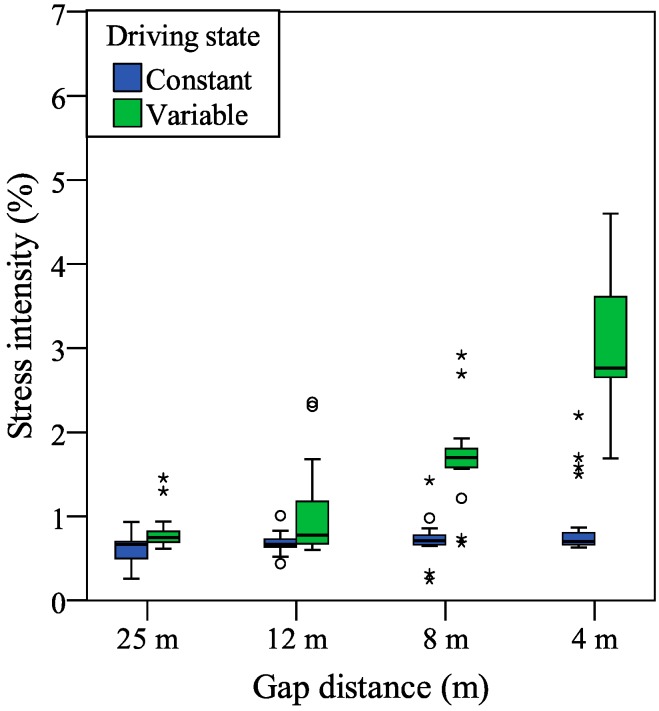
Stress intensity estimated from palmar perspiration. Depending on the interquartile range for the box plot, outliers are indicated by circles (1.5–3) or stars (>3).

The results of the stress intensity estimated from palmar perspiration are presented in Figure 8. Related to statistical analysis, a two-way repeated measures ANOVA was adopted with two variables (driving state × gap distance). As the within-subject factor, one variable is related with the driving state: constant and variable, and another is related with the gap distance: 25 m, 12 m, 8 m, and 4 m.

For the stress intensity estimated from palmar perspiration, there were a significant main effect of the driving state (*F*[1, 19] = 71.72, *p* < 0.001, partial *η*^2^ = 0.79), indicating that the mean stress intensity was significantly higher for the variable condition (*M* = 1.79, *SD* = 0.13) than for the constant condition (*M* = 0.73, *SD* = 0.03), and a significant main effect of the gap distance (*F*[3, 57] = 24.86, *p* < 0.001, partial *η*^2^ = 0.58), indicating that the mean stress intensity was significantly different for the gap distances of the 25 m (*M* = 0.71, *SD* = 0.03), 12 m (*M* = 0.85, *SD* = 0.06), 8 m (*M* = 1.21, *SD* = 0.06), and 4 m (*M* = 2.26, *SD* = 0.27). Additionally, a significant interaction was found between the driving state and gap distance (*F*[3, 57] = 30.29, *p* < 0.001, partial *η*^2^ = 0.61).

Furthermore, by a pairwise comparison with Bonferroni correction, there was a significant difference between the stress intensities of the constant and variable driving states (*p* < 0.001). For the stress intensities in the different gap distances, Bonferroni-corrected *post hoc* tests showed that, the stress intensity for the 4 m gap distance was significantly higher than that of the 8 m, 12 m, and 25 m gap distances (*p* < 0.001, *p* < 0.001, and *p* < 0.01); the stress intensity for the 8 m gap distance was also significantly higher than that of the 12 m and 25 m gap distances (both *p* < 0.001); however, there was no significant difference between the stress intensities of the 12 m and 25 m gap distances (*p* = 0.24).

**Figure 9:**
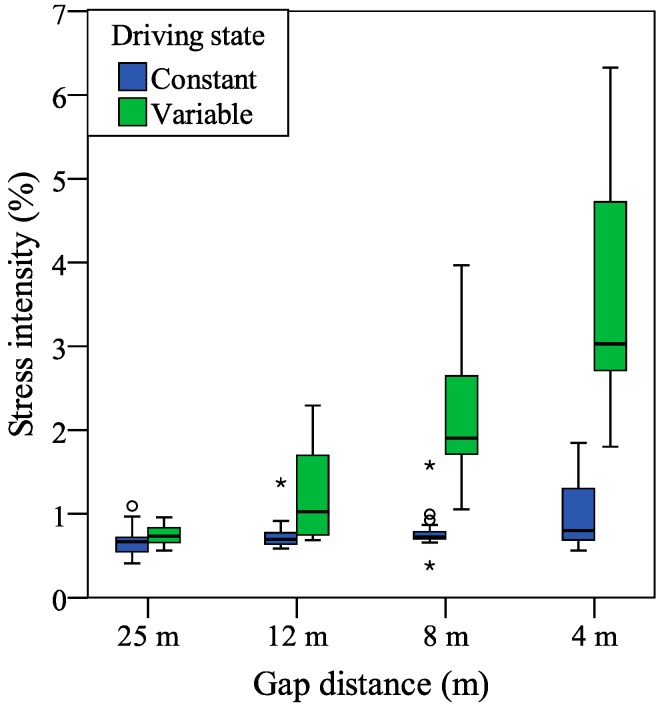
Stress intensity estimated from masseter EMG signal. Depending on the interquartile range for the box plot, outliers are indicated by circles (1.5–3) or stars (>3).

The results of the stress intensity estimated from masseter EMG signal are presented in Figure 9. Related to statistical analysis, a two-way repeated measures ANOVA was also processed with two variables (driving state × gap distance).

For the stress intensity estimated from masseter EMG signal, there were a significant main effect of the driving state (*F*[1, 19] = 116.20, *p* < 0.001, partial *η*^2^ = 0.86), indicating that the mean stress intensity was significantly higher for the variable condition (*M* = 1.95, *SD* = 0.12) than for the constant condition (*M* = 0.79, *SD* = 0.04), and a significant main effect of the gap distance (*F*[3, 57] = 64.20, *p* < 0.001, partial *η*^2^ = 0.77), indicating that the mean stress intensity was significantly different for the gap distances of the 25 m (*M* = 0.70, *SD* = 0.02), 12 m (*M* = 0.98, *SD* = 0.07), 8 m (*M* = 1.51, *SD* = 0.10), and 4 m (*M* = 2.29, *SD* = 0.16). Additionally, a significant interaction was found between the driving state and gap distance (*F*[3, 57] = 59.75, *p* < 0.001, partial *η*^2^ = 0.76).

Furthermore, by a pairwise comparison with Bonferroni correction, there was a significant difference between the stress intensities of the constant and variable driving states (*p* < 0.001). For the stress intensities in the different gap distances, Bonferroni-corrected *post hoc* tests showed that, the stress intensity for the 4 m gap distance was significantly higher than that of the 8 m, 12 m, and 25 m gap distances (all *p* < 0.001), the stress intensity for the 8 m gap distance was significantly higher than that of the 12 m and 25 m gap distances (both *p* < 0.001), and the stress intensity for the 12 m gap distance was significantly higher than that of the 25 m gap distances (*p* < 0.01).

### 3.3. Subjective Evaluation

**Figure 10:**
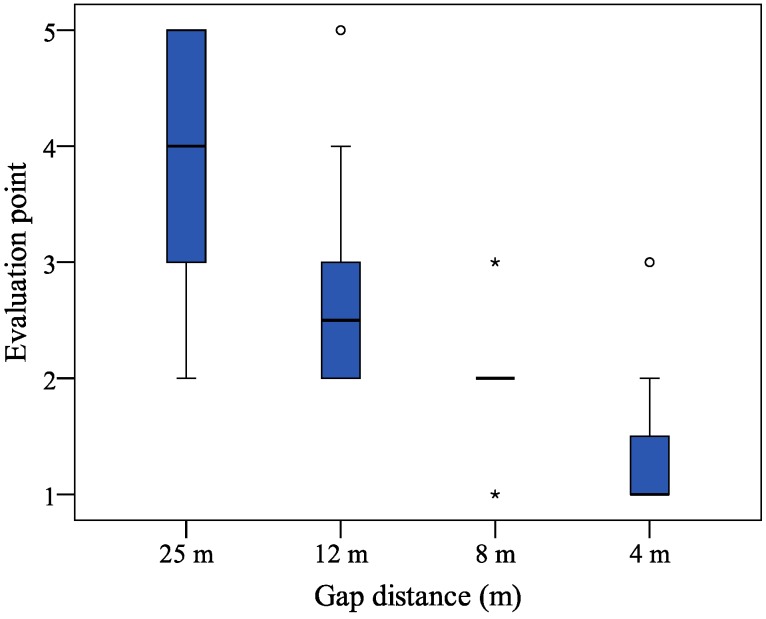
Subjective evaluation of ride comfort for the different driving conditions. Depending on the interquartile range for the box plot, outliers are indicated by circles (1.5–3) or stars (>3).

The subjective evaluation of ride comfort was investigated and the results of the evaluation points (which could range from 1 to 5) are presented in Figure 10. A one-way nonparametric Friedman ANOVA was conducted at the 0.05 level of significance. There was a significant main effect (*F*_r_[3, 20] = 50.0, *p* < 0.001), indicating that the evaluation points of ride comfort significantly changed over the four experimental conditions of the 25 m gap distance (*M* = 3.8, *SD* = 1.0), 12 m gap distance (*M* = 2.8, *SD* = 0.9), 8 m gap distance (*M* = 1.9, *SD =* 0.6), and 4 m gap distance (*M* = 1.3, *SD* = 0.6). The results indicate that the participants felt significant discomfort as the gap distance decreased.

In the 4 and 8 m gap distances in the automatic driving condition, most of the participants reported that they felt discomfort and little discomfort, respectively. In the 12 m gap distance in the automatic driving condition, half of the participants reported feeling little discomfort, while the other scores were mixed, with others reported the experience was ‘normal’ or provided ‘little comfort’. In the 20–30 m gap distance in the manual driving condition, most participants reported comfort, while the others reported that the driving experience was ‘common’ or provided ‘little comfort’.

## 4. Conclusions

For the purpose of identification of driver state during automatic driving of trucks, mental stress of drivers was quantitatively evaluated through biosignal analyses in a driving simulator experiment. The palmar perspiration and masseter EMG signals are distinct, but both of them were continuously measured by application of two wearable measurement apparatuses, respectively. On the hypothesis that the closer and variable gap distance may result in higher mental stress of drivers, ten participants tested the four driving conditions: manual driving with 20–30 m gap distance, and automatic driving with 4, 8, and 12 m gap distance.

A significant main effect for the gap distance factor indicates that the mean mental stress intensity was significantly higher for the closer gap distances, and a significant main effect for the driving state factor interprets that the mental stress intensity was significantly higher for the variable gap distance than for the constant gap distance. In conclusion, the mental stress of drivers significantly increased as the gap distances decreased, and an especially abrupt increase in mental stress was observed during sudden deceleration variation in automatic driving of trucks. Subjective reports indicated that this increase in stress was associated with a significantly increase in driver discomfort.

The statistical analyses from the two measured biosignals revealed identical tendency with respect to mental stress of drivers; however, by a pairwise comparison with Bonferroni correction between the 12 and 25 m gap distances, the significant differences are different for the stress intensity estimated from palmar perspiration and those from masseter EMG signal. It was considered that the stress intensity in the variable driving state always sharply increased with the closer gap distance; however, the stress intensity in the constant driving state always gradually increased with the closer gap distance, especially from the 25 to 12 m gap distance. Therefore, even though the stress intensity for the 12 m gap distance was significantly higher than that of the 25 m gap distance in the variable driving, the stress intensities between the 12 and 25 m gap distances remained at approximately the same level in the constant driving state, especially for the palmar perspiration.

In this study, the driving experiment was only conducted with ten healthy male drivers. Therefore, for a wider implementation, further investigation should consider a larger representative sample of drivers from different gender and age groups. Furthermore, although researchers have begun to consider human factors to design intelligent vehicle systems, the relevant problems remain poorly understood [31]. A further insight into human-machine interactions can benefit further understanding of driver-vehicle behaviors in complicatedly automatic environments. Out of question, a successful application of automatic technology is dependent on the improvement of user acceptability, which also requires a substantial understanding of human-machine interactions [32]. In the near future, it becomes necessary to design and evaluate a human-machine interface for alleviating the mental stress of drivers to improve user acceptability.
